# Feasibility study of deep learning‐based markerless real‐time lung tumor tracking with orthogonal X‐ray projection images

**DOI:** 10.1002/acm2.13894

**Published:** 2022-12-28

**Authors:** Dejun Zhou, Mitsuhiro Nakamura, Nobutaka Mukumoto, Yukinori Matsuo, Takashi Mizowaki

**Affiliations:** ^1^ Department of Advanced Medical Physics Graduate School of Medicine Kyoto University Kyoto Japan; ^2^ Department of Radiation Oncology and Image‐Applied Therapy Graduate School of Medicine Kyoto University Kyoto Japan; ^3^ Department of Radiation Oncology Graduate School of Medicine, Osaka Metropolitan University Osaka Japan

**Keywords:** deep learning, markerless real‐time tumor tracking, respiratory motion management, target contour prediction

## Abstract

**Purpose:**

The feasibility of a deep learning‐based markerless real‐time tumor tracking (RTTT) method was retrospectively studied with orthogonal kV X‐ray images and clinical tracking records acquired during lung cancer treatment.

**Methods:**

Ten patients with lung cancer treated with marker‐implanted RTTT were included. The prescription dose was 50 Gy in four fractions, using seven‐ to nine‐port non‐coplanar static beams. This corresponds to 14–18 X‐ray tube angles for an orthogonal X‐ray imaging system rotating with the gantry. All patients underwent 10 respiratory phases four‐dimensional computed tomography. After a data augmentation approach, for each X‐ray tube angle of a patient, 2250 digitally reconstructed radiograph (DRR) images with gross tumor volume (GTV) contour labeled were obtained. These images were adopted to train the patient and X‐ray tube angle‐specific GTV contour prediction model. During the testing, the model trained with DRR images predicted GTV contour on X‐ray projection images acquired during treatment. The predicted three‐dimensional (3D) positions of the GTV were calculated based on the centroids of the contours in the orthogonal images. The 3D positions of GTV determined by the marker‐implanted RTTT during the treatment were considered as the ground truth. The 3D deviations between the prediction and the ground truth were calculated to evaluate the performance of the model.

**Results:**

The median GTV volume and motion range were 7.42 (range, 1.18–25.74) cm^3^ and 22 (range, 11–28) mm, respectively. In total, 8993 3D position comparisons were included. The mean calculation time was 85 ms per image. The overall median value of the 3D deviation was 2.27 (interquartile range: 1.66–2.95) mm. The probability of the 3D deviation smaller than 5 mm was 93.6%.

**Conclusions:**

The evaluation results and calculation efficiency show the proposed deep learning‐based markerless RTTT method may be feasible for patients with lung cancer.

## 1 INTRODUCTION

Respiratory motion management is vital during radiation therapy for patients with lung tumors.^1^ The internal target volume (ITV) method is one of the most commonly used approaches. This method covers the range of tumor motion by enlarging the target volume.^2^ But the enlarged target volume may increase the dose to healthy tissue, and a previous study showed that the ITV method may not fully cover the tumor motion range during beam delivery.^3^ Abdominal compression, breath‐hold, and respiratory gating can also reduce the effects of tumor motion.^4^ But these approaches may cause burdens to the patient's breath or prolong the treatment duration.

A more advanced approach to achieve respiratory motion management is real‐time tumor tracking (RTTT).^5^ By tracking the target position while a patient breathes freely, the dose to healthy tissue can be controlled without adding an extra burden to the patient during treatment beam delivery.

Currently, caused by the limited soft‐tissue contrast on X‐ray images, the marker‐implanted approach is mainly adopted to assist X‐ray image based RTTT. Metallic fiducial markers are implanted into the patient's lung around the tumor approximately 1 week prior to the planning computed tomography (pCT) scan.^6^ By detecting the internal markers, the position of the target can be determined using the geometric correlation of markers’ centroid and target in the pCT. After years of clinical practice, the shortcomings of the marker‐implanted approach have been realized.^7^ The implantation of markers is an invasive procedure. It prolongs the total treatment duration by approximately one week and may cause implantation‐related complications. As the markers are implanted before the pCT scan and near the target, metal artifacts may appear and blur the pCT image, especially in the area around the target.^8^ Marker induced error caused by inter‐ and intra‐fractional marker migration has also been reported.^9^ The migration of markers may cause changes in geometric correlation and decrease the accuracy of marker‐implanted RTTT.^7^

To overcome the shortcomings of the marker‐implanted approach, markerless RTTT is required. Markerless RTTT is defined as a real‐time tumor positioning system in which no exogenous materials are implanted into the patient body to assist the image‐guiding process.^10^ Without the implantation of markers, the total treatment duration can be shortened, and patients will be free from potential risks. With the markerless approach, metal artifacts in pCT and marker‐induced errors can be efficiently eliminated.

Several commercial and phantom studies on markerless RTTT have recently reported the potential advantages of this approach. These studies can be categorized as template‐based.^11^, ^12^ and deep learning‐based.^13^, ^14^ Template‐based methods track the target by generating target templates from digitally reconstructed radiograph (DRR) images and matching the template on the kV X‐ray projection images during the treatment. Template‐based approaches are rigid and commonly adopted in commercial software, such as CyberKnife's XSightLung (Accuray, Sunnyvale, CA).^11^ and RapidTrack (Varian Medical Systems, Palo Alto, CA).^12^ Both studies reported limitations. XSightLung cannot track lung tumors with a diameter small than 15 mm.^11^ For small lung tumors, RapidTrack can only track tumor with limited motion.^12^ Deep learning‐based approaches use target‐labeled DRR images to train a deep learning model. Takahashi et al. developed a deep learning model to predict the bounding box of spherical and ovoid target in phantom on X‐ray projection images.^13^ Previous work used a deep learning model to predict the contour of the pancreas tumor's clinical target volume on DRR images.^14^ The position of the target is determined by the center of the target bounding box or the centroid of the target contour. Although previous deep learning‐based studies provided accurate results, the images for evaluation were from phantom studies or synthetic images, which made it doubtful whether the accuracy can be maintained in clinical practice with real patients and treatment beam irradiation. Currently, for marker‐implanted RTTT for patients with lung cancer, the margin between planning target volume and GTV was at least 8 mm on one direction.^15^ Clinically, for markerless RTTT, a three‐dimensional (3D) tracking accuracy better than 5 mm will bring the technology a bright future.

In the prior study, the deep learning‐based method was trained and evaluated on DRR images^14^; however, the feasibility of the method is unclear if evaluated with the real X‐ray projection images acquired during treatment. As far as we know, there was no published work about deep learning‐based markerless RTTT method evaluated with X‐ray projection images and clinical tracking record acquired during patient's treatment. In the current study, the deep learning‐based markerless RTTT procedure was trained on DRR images and tested using orthogonal X‐ray projection images acquired from real patient treatments. To the best of our knowledge, the present study is the first to perform dynamic gross tumor volume (GTV) contour prediction on real X‐ray projection images and evaluate the performance of tracking with 3D target position data acquired during treatment. The data generation and training of the deep learning‐based target contour prediction model are patient‐ and kV X‐ray tube angle‐specific. The data generation consists of DRR generation from four‐dimensional CT (4DCT) and data augmentation. The data augmentation method can generate sufficient data for model training. Thus, there is no need to accumulate historical data. During testing of the procedure, the GTV contour of the lung tumor in the real X‐ray projection image was predicted, and the 3D position of the tumor was calculated based on the centroids of the orthogonal GTV contours on real X‐ray projection images. The 3D tracking accuracy of the procedure was then evaluated and analyzed with the record acquired during treatment, which made this feasibility study more clinically realistic than previous phantom studies.

## 2 MATERIALS AND METHODS

### 2.1 Marker implanted real‐time tumor tracking of Vero4DRT system

The Vero4DRT system (Hitachi Ltd., Tokyo, Japan and Brainlab AG, Feldkirchen, Germany) has a gimbaled 6 MV X‐ray head and an orthogonal kV X‐ray imaging system.^16^ The gimbaled‐head and imaging system were installed with a compact accelerator in an O‐ring gantry. The O‐ring gantry can rotate ± 60^°^ about its vertical axis and ± 185^°^ along its O‐shaped structure. The imaging system rotates simultaneously with the gantry and O‐shaped structure. The orthogonal kV X‐ray imaging system consisted of two pairs of X‐ray tubes and flat‐panel detectors (FPDs). The X‐ray source to isocenter distance and FPD distance were 1000 and 1836 mm, respectively. The maximum size of the FPDs at the isocenter level was 222 mm × 168 mm, with a spatial resolution of 0.211 mm at the isocenter level. The FPD pixel array size is 1024 × 768.

The imaging system works with an extended version of the ExacTRAC system (Brainlab AG) to perform 3D tumor tracking.^17^ For patients with lung cancer, 2 to 5 disposable gold markers (Olympus Medical Systems, Tokyo, Japan) were implanted before patient's 4DCT scan. The system detects implanted fiducial markers on X‐ray images and calculates the centroid of the marker polyhedron. With the marker centroids on the orthogonal X‐ray images, the 3D markers center of mass was defined as the midpoint of the shortest intersection vector between the vectors connecting the orthogonal kV X‐ray source and centroid of the corresponding markers on the FPDs. Clinically, the detected GTV position is continuously determined with the 3D marker center of mass plus the relative shift between the center of mass of markers and the GTV at the reference phase. The reference phase was the selected phase of the patient's 4DCT.

### 2.2 Patient characteristics and treatment planning

This retrospective study was approved by the institutional review board (R1446). The phase‐based 4DCT scans of ten patients with lung cancer were included in this study (Table 1). CT simulations were performed using the BodyFix system (Elekta AB, Stockholm, Sweden) with overhead‐raised arms. The 4DCT scans were acquired using a LightSpeed RT 16‐slice CT simulator (General Electric Medical Systems, Waukesha, WI) or SOMATOM Definition AS (Siemens Medical Systems, Erlangen, Germany), with a real‐time positioning management system (Varian Medical System). The CT slice thickness was 2.5 mm for patients 1 to 6 and 2 mm for others. The entire respiratory period was divided into 10 respiratory phases. The radiation oncologist contoured the GTV for each phase.

**TABLE 1:** Patient characteristics

Patient index	Sex	Age [y]	Location	Stage	** GTV** volume [cm^3^]	** Mean HU value of GTV**	3D motion range [mm]	Number of markers
1	M	86	Right middle lobe	cT1bN0M0	7.0	–176.2	22	3
2	M	88	Right middle lobe	cT1bN0M0	11.6	–260.0	28	4
3	F	90	Left lower lobe	cT1aN0M0	5.1	–145.7	13	4
4	M	82	Left upper lobe	cT1bN0M0	6.0	–203.9	25	3
5	F	83	Left lower lobe	cT1bN0M0	15.8	–340.0	22	2
6	M	71	Right lower lobe	cT1bN0M0	7.9	–329.6	26	4
7	M	81	Right upper lobe	cT1bN0M0	2.8	–249.7	11	5
8	M	79	Right lower lobe	cT2aN0M0	25.7	–19.8	25	4
9	M	79	Right lower lobe	metastasis	1.2	–346.0	13	5
10	F	71	Right lower lobe	cT1cN0M0	17.8	–37.7	17	4

Abbreviations: 3D, three‐dimensional; F, female; GTV, gross tumor volume; HU, Hounsfield unit; M, male.

All patients underwent seven to nine‐port non‐coplanar 3D conformal radiation therapy (3DCRT) with RTTT. Correspondingly, the clockwise and counterclockwise X‐ray tube angles were gantry angles plus and minus 45^°^, respectively. The prescription dose was 50 Gy in four fractions.

### 2.3 Generation of training dataset

For a deep learning model, sufficient data are required to train the model. This section presents a data augmentation method aimed at generating sufficient labeled data to train a patient and X‐ray angle‐specific deep learning model.

**FIGURE 1:**
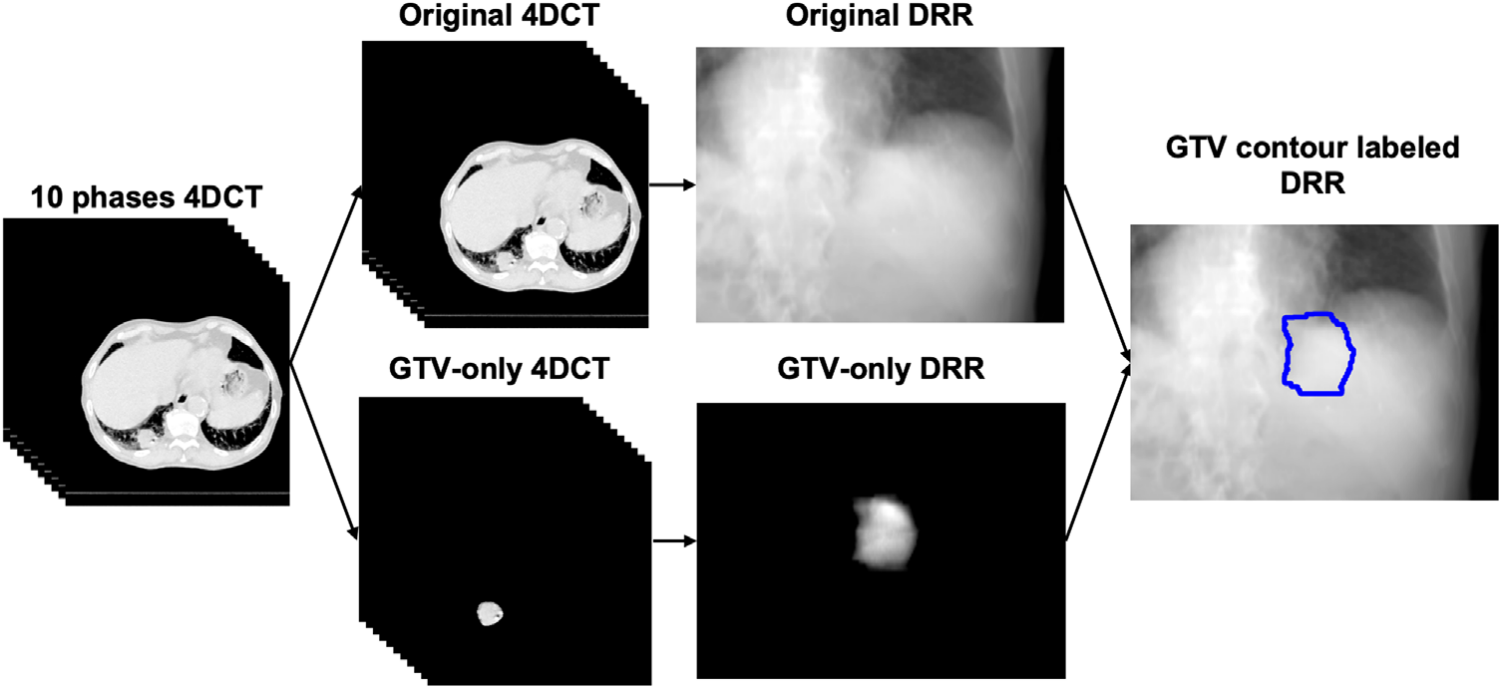
Overall workflow of training dataset generation

The overall workflow of the training dataset generation is shown in Figure 1. The raw data was a patient 4DCT, which contained CT volumes of 10 respiratory phases. GTV‐only CT volumes were acquired by extracting the GTV contour from the DICOM structure storage file using Python. Subsequently, the open‐source program Plastimatch was applied to generate DRR images for both the original and corresponding GTV‐only CT volumes.^18^ Plastimatch generates DRR images by using Siddon ray tracing method.^19^ Scatters were not considered during DRR images generation. The geometric settings for DRR generation were obtained from the technical description of Vero4DRT system. As a result, ten paired original DRR and GTV‐only DRR images were acquired.

The first step of data augmentation was to rotate the X‐ray beam angle around the isocenter from ‐3.5^°^ to 3.5^°^ in 0.5^°^ intervals in both the superior‐inferior and anterior‐posterior directions for DRR image generation. As the angle was rotated 15 times for each direction, the data scale was augmented 225‐fold after this step. Thus, 2,250 paired original DRR and GTV‐only DRR images were acquired.

The second step was to extract the mask of the GTV from the GTV‐only DRR images and overlap it with the corresponding original DRR images to obtain the GTV contour‐labeled DRR. For each X‐ray angle for a patient, 2,250 GTV contour labeled DRR images were used to train the patient and an X‐ray angle‐specific deep learning model. The scale of the training data was considered sufficient by referring to previous work.^14^

### 2.4 Patient and X‐ray angle‐specific deep learning model for target contour prediction

**FIGURE 2:**
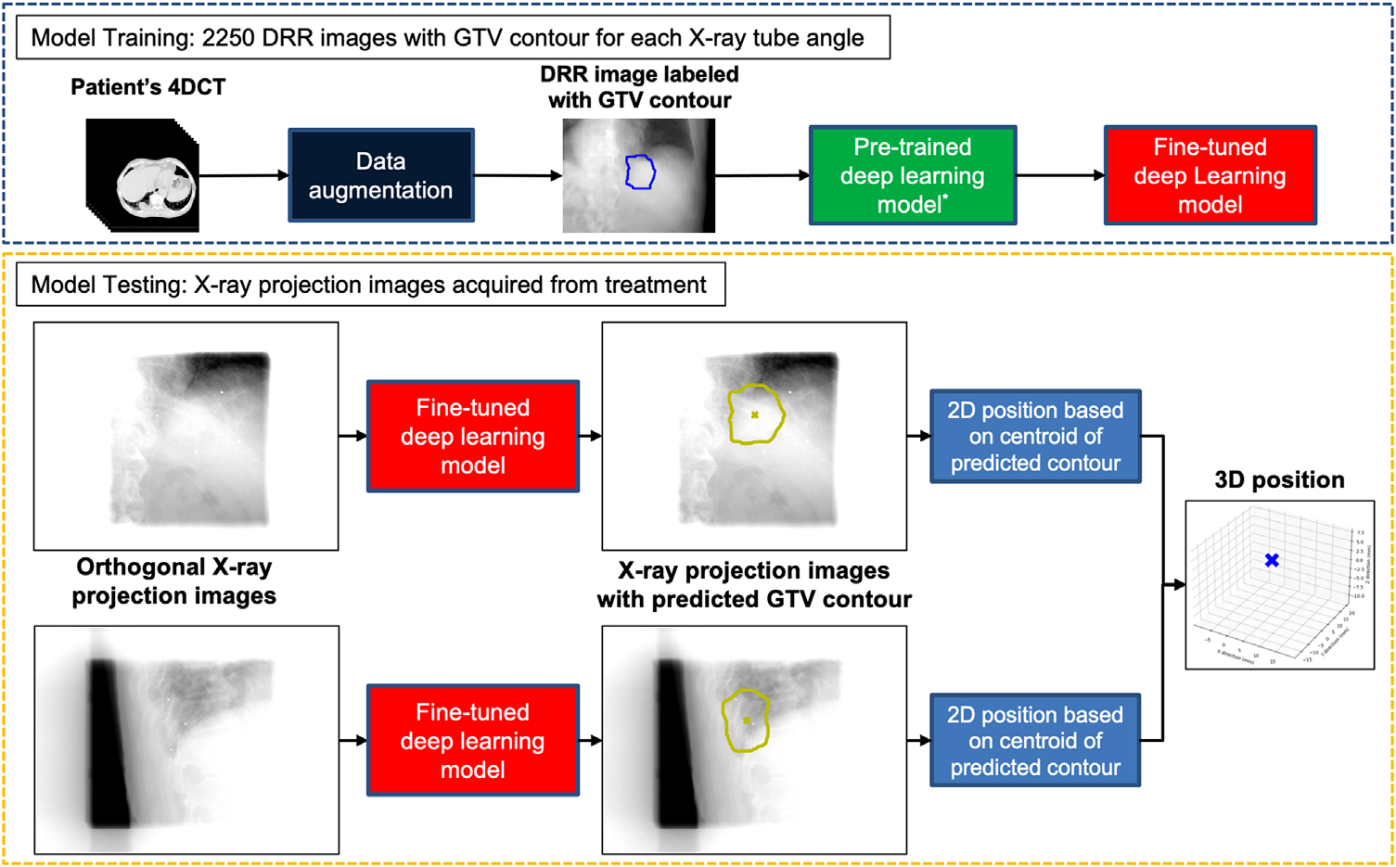
Workflow for training and testing a patient and X‐ray angle‐specific deep learning model for target contour prediction

The workflow for training and testing a patient and an X‐ray angle‐specific deep learning model for target contour prediction are shown in Figure 2. The target of this study was the GTV. In brief, this model can predict the contour of the target in each region of interest (RoI).^20^ with features extracted by ResNet.^21^ and a feature pyramid network.^22^ The loss function contains loss on classification, bounding box.^23^ and contour.^20^ The deep learning framework was detectron2.^24^ and the model was pretrained on the COCO dataset.^25^ Then, the pre‐trained model was fine‐tuned to predict the GTV contour with 2,250 GTV contour labeled DRR images, which were generated in the previous step. Subsequently, a GTV contour was used to predict the deep learning models for each corresponding X‐ray tube angle.

### 2.5 Performance evaluation

During the performance evaluation, the models trained with DRR images were tested with real X‐ray projection images acquired during treatment. The projection images were processed with a Gaussian filter to simulate the same spatial resolution as the DRR images, and then imported into the patient and X‐ray angle‐specific deep learning model. Projection images were acquired during treatment beam delivery with a collimated kV beam field size to reduce the imaging dose. The deep learning model predicted the GTV contours in the projection images. Subsequently, the centroid of the predicted contour is calculated. The 3D predicted positions can be determined using centroids on the orthogonal projection images. The method used to calculate the 3D predicted GTV position was the same as that used in the Vero4DRT system, which calculates the position of 3D markers center of mass.

The centroids of the markers were recorded during the treatment, and the 3D GTV positions were determined. In this study, 3D GTV positions determined by the clinical records were considered as the ground truth. The 3D deviation between the ground truth and the 3D predicted GTV position was calculated to evaluate the performance of the model for tumor positioning. Pearson correlation coefficient (*r*) was calculated to evaluate 3D deviation and prediction rate dependent on GTV volume, GTV motion range, and fraction index.

## 3 RESULTS

The calculation efficiency of the deep learning model can meet the requirement of clinical practice. The high‐performance computer (HPC) used in this study had an Intel Core i7 9800X central processing unit (CPU), four Quadro GV100 32 GB graphics processing units (GPUs), and eight 16 GB RAM modules. The training time for one GPU was 2.5 s per iteration. For 2,000 iterations, the training time for one X‐ray tube angle of a patient was approximately 1.5 h. As the HPC had four GPUs and each patient had 14–18 X‐ray tube angles, the total training time for one patient was approximately 6 h. The calculation time for gaussian filter with CPU was about 30 ms. The average GTV contour prediction time with one GPU for one image was around 55 ms. The total calculation time per image was about 85 ms. Because the orthogonal images can be calculated in parallel on two GPUs, the calculation efficiency is sufficient for RTTT.

For all the patients and fractions, 15,140 pairs of orthogonal X‐ray projection images were stored. The marker‐implanted method recorded 13,244 3D positions (87.5%). The proposed markerless procedure predicted 10,242 3D positions (67.6%). Finally, 8993 pairs of images could be detected by the marker‐implanted method and predicted by the markerless procedure. These 8993 comparisons were included in the statistical results of performance evaluation.

**FIGURE 3:**
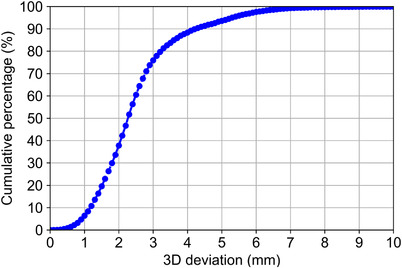
Cumulative percentage curve of 3D deviation between the predicted position and ground truth

The overall median value of the 3D deviation was 2.27 (interquartile range [IQR]: 1.66–2.95) mm. Figure 3 shows the cumulative percentage curve of the 3D deviation. The percentage of 3D deviation smaller than 3 and 5 mm was 75.9% and 93.6%, respectively.

Tables 2 and 3 show the 3D deviation and prediction rate of each patient, and the 3D deviation and prediction rate of each fraction, respectively. Notably, patient number 2 had much worse predication accuracy and prediction rate than the other patients. In the calculation of Pearson correlation coefficients, the results excluded patient number 2 were also calculated to prevent potential bias. The calculation results of Pearson correlation coefficient are shown in Table 4.

**TABLE 2:** 3D deviation and prediction rate regarding to each patient

Patient index	Median of 3D deviation (IQR) [mm]	Prediction rate [%]
1	2.45 (2.02–2.89)	78.5
2	5.65 (5.07–6.36)	24.0
3	2.48 (1.87–3.29)	68.0
4	2.48 (2.27–2.84)	96.0
5	1.81 (1.44–2.15)	98.0
6	3.17 (2.70–3.81)	61.7
7	1.75 (1.43–2.13)	46.6
8	1.56 (1.00–2.65)	72.1
9	1.94 (1.43–2.54)	42.0
10	2.39 (1.54–3.54)	98.8
Overall	2.27 (1.66–2.95)	67.6

Abbreviation: IQR, interquartile range.

**TABLE 3:** 3D deviation and prediction rate regarding to each fraction

Fraction index	Median of 3D deviation (IQR) [mm]	Prediction rate [%]
1	2.16 (1.52–2.99)	75.5
2	2.17 (1.64–2.75)	65.2
3	2.36 (1.83–2.90)	65.7
4	2.42 (1.74–3.23)	64.2
Overall	2.27 (1.66–2.95)	67.6

Abbreviation: IQR, interquartile range.

**TABLE 4:** Pearson correlation coefficient calculation results

	GTV volume	3D motion range	Fraction index
Median 3D deviation	–0.19 (–0.15)	0.52 (0.31)	0.95 (0.98)
Prediction rate	0.38 (0.53)	0.09 (0.51)	–0.82 (–0.92)

*Note*: Only the values in parentheses were adapted in the discussion to prevent bias. The values in parentheses are the *r* values without patient number 2.

Abbreviations: 3D, three‐dimensional; GTV, gross tumor volume.

## 4 DISCUSSION

The calculation time was about 85 ms per image. It is much smaller than the tolerance of the total system latency for RTTT recommended by AAPM TG‐76, which was 500 ms. We realized that contour prediction is a part of the total system latency. Because the stored data was adopted, it was difficult to include other components. Further phantom studies may include data acquisition time, data transformation time and optimization of program to better evaluate the total system latency of markerless tumor tracking.

The causes of 3D deviation may lie in both the proposed procedure and ground truth. For this procedure, the target contour detection model was fine‐tuned with DRR images and tested with real X‐ray projection images. Compared to real projection images, the sharpness of the DRR images was poor, and the contrast was different. Meanwhile, because the real projection images were recorded with the treatment beam delivery, there was noise caused by megavoltage scatters in the real projection images [26]. The noise and difference between the training and testing images may decrease the accuracy of the target contour prediction model. Regarding the ground truth, clinically before the RTTT, the patient setup error correction was based on the bone anatomy, not the GTV. Based on an unpublished institutional discussion, by comparing planning CT before radiation therapy and cone‐beam CT after each fraction for 145 patients who underwent lung stereotactic body radiation therapy, a systematic shift of approximately 2 mm in 3D between the GTV and bone structure was observed. This may also have contributed to the deviations.

**FIGURE 4:**
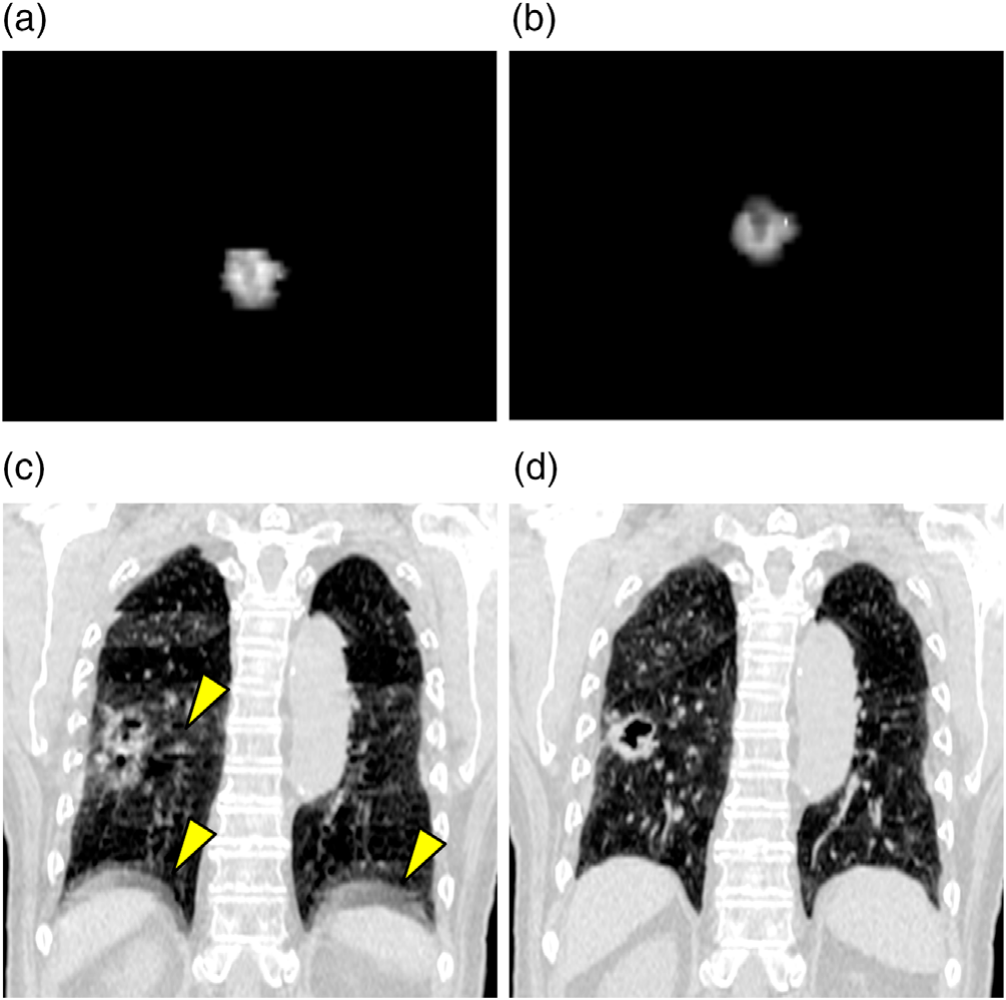
Demonstration of artifacts and inconsistent GTV contours on DRR images and CT volume of Patient 2. (a) DRR at end of inhalation, X‐ray tube angle at 275°; (b) DRR at end of exhalation; X‐ray tube angle at 275°; (c) Coronal view of CT at end of inhalation, yellow arrows indicate artifacts; (d) Coronal view of CT at end of exhalation

For patient numbers 7, 8, 9, and 10, the prediction accuracy was relatively better than other patients. One of the possible reasons may be related to the CT slice thickness. For these four patients, the slice thickness was 2 mm, while it was 2.5 mm for the rest. Thinner CT slice thickness made the DRR images more similar to the real X‐ray projection images, which enabled the deep learning model to be better trained and provided better results. As shown in Table 2, the 3D deviation of patient number 2 was significantly larger while the prediction rate was much lower than the others. For this patient, by comparing Figure 4a,b, inconsistent GTV shapes on DRR images were observed. This was caused by severe 4DCT artifacts, as indicated by yellow arrows in Figure 4c and comparing with Figure 4d. The 4DCT artifacts caused the DRR images and GTV contours for training to be different from the real X‐ray projection images and GTV, and the markerless procedure provides relatively poor results.

To prevent bias in the analyze of correlations, only the *r* values without patient number 2 were discussed in this paragraph. As shown in Table 4, the median 3D deviation has no correlation with GTV volume (*r* = ‐0.15), and weak correlation with motion range (*r* = 0.31). A strong correlation between fraction index and median 3D deviation (*r* = 0.98) was observed. The strong correlation may be caused by the change in the GTV shape compared to the day of the 4DCT scan as the treatment progressed. Regarding the ground truth side, interfractional marker migration may also cause the 3D deviation to increase as the treatment progresses.^9^

The prediction rate of the proposed procedure was 67.6%. It was lower than the detection rate of the marker‐implanted method, which was 87.5%. There may be three main reasons that limit the current prediction rate. First was the fact that the imaging parameters and X‐ray angles were adjusted for marker detection for each patient.^27^ Second was to reduce the imaging dose to the patient.^28^ the field of view for the kV X‐ray tube was also narrowed by the on‐site medical physicist. These protocols resulted in the X‐ray projection images that differ from the DRR image and further reduced the detection rate. Third was as shown in Table 4. The correlations between prediction rate and GTV volume (*r* = 0.53), and between prediction rate and motion range (*r* = 0.51) were not strong. Meanwhile a strong negative correlation with fraction index was observed (*r* = ‐0.92), indicating that the prediction accuracy decreased with treatment progressing. This may be also caused by the change in the GTV shape and its surroundings compared to the day of the 4DCT scan.

Comparing with the template‐based markerless tracking, the proposed deep learning‐based procedure showed superior performance on small targets. Bahig et al. reported that with the XSightLung system, the prediction rate was 66% for targets diameter larger than 15 mm.^11^ In the present study, the overall detection rate was 67.6%, without a preselection based on favorable characteristics. Remmerts de Vries et al. reported a study using the RapidTrack system.^12^ In their work, projection images were acquired without treatment beam delivery, and preselection was conducted based on target motion range (1–8 mm, longitudinal 4DCT tumor motion range). The prediction rate of their work was 71% and predicted position was within a 5 mm PTV margin for 95.5% of the time. In the present study, the detection rate was 67.6%, and the percentage of 3D deviation within 5 mm of the detected GTV position was 93.6%. Considering that the present study contained targets with a larger motion range and projection images were acquired with MV beam delivery, the presented deep learning‐based procedure is applicable to a larger spectrum of patients and has more prospects for clinical application.

For comparison with deep learning‐based approaches, Takahashi et al. only used a phantom with spherical or ovoid targets.^13^ On the X‐ray projection images of the phantom studies, there were no MV scatters, and the shapes of the targets were spherical. Zhou et al. only evaluated with synthetic images.^14^ In comparison to these studies, this study used data from real patient treatments. In this study, the shape of the GTV was manually contoured and X‐ray projection images were recorded during treatment with MV beam delivery. Meanwhile, in phantom studies, structures other than the target were stable, and no anatomical change with respiration motion was simulated. In the present study, the images were obtained from the free breathing of real patients, which made the results of this study more reliable, and the presented procedure was closer to the clinical practice. The fact that the accuracy of tracking in the previous phantom studies was better than that in the present study was acknowledged. However, if a comparison is made with prior research.^14^ which used the same model to predict the clinical target volume of patients with pancreatic tumors on DRR images, it can be concluded that the present study may be superior when treating real patients.

This study may influence the current clinical practice in three aspects. First, as there will be no need to implant markers into the patient body, the total treatment duration may be shortened by about 7 days, and patients will be free from the potential risk. This may greatly benefit patients. Second, with the deep learning‐based markerless procedure, the margin of the target can be narrowed. For the proposed work, the GTV contour was predicted directly, and the percentage of 3D deviation smaller than 5 mm was 93.6%. It may be reasonable to reduce the PTV margin because the marker‐induced error was diminished. Shrinkage of the margin may significantly reduce the dose to healthy organs, thus further improving the patient quality of life and avoiding radiation toxicity. Third, the proposed method may be applicable for volumetric modulated arc therapy in the future. To achieve this, training datasets consisting of DRR images for a designated angle step needs to be generated. Then for each angle step, deep learning model is trained and then predict GTV contour on X‐ray projection images at the angle step during treatment.

Three limitations of this study were recognized. The first limitation may be the presence of markers. A previous study done by Zhao et al. showed that for deep learning model, the presence of markers doesn't impact on the performance of model.^29^ In this study, the diameter of implanted marker was 1.5 mm. The volume of markers was small and may not impact the performance of the model. The second limitation was the detection rate. Although the presented procedure will shorten the total treatment duration, a lower detection rate may prolong the duration per fraction. As discussed previously, historical X‐ray projection images were acquired based on parameters adjusted for marker detection for each patient, while all DRR images were generated with the same parameters. Further phantom studies may remove the presence of markers, set consistent parameters and protocols optimized to solve the above two limitations. The third limitation was that the 4DCT quality and GTV contour consistency may greatly affect the accuracy of contour prediction, as the results of patient number 2 indicated. For the preparation of markerless RTTT before radiation therapy, protocols for 4DCT scanning and GTV contouring are required to ensure accurate contour prediction during radiation therapy.

## 5 CONCLUSION

In this study, the feasibility of a deep learning‐based markerless RTTT with orthogonal X‐ray projection images for patients with lung tumors was presented. Ten patients treated by seven to nine non‐coplanar 3DCRT with RTTT were included. A total of 8,993 3D positions between the predicted position and ground truth were compared. The overall median value of the 3D deviation was 2.27 (IQR: 1.66–2.95) mm. The probability for 3D deviation smaller than 3 and 5 mm was 75.9% and 93.6%, respectively. The calculation time per image was about 85 ms.

The evaluation results and calculation efficiency show the deep learning‐based markerless RTTT method may be feasible for patients with lung cancer. Future studies may focus on thorax phantom studies without markers and with real tumor shape and motion to further refine the markerless tracking procedure.

## AUTHOR CONTRIBUTIONS

Dejun Zhou and Mitsuhiro Nakamura planned the study, performed the statistical analysis, and drafted the manuscript. Nobutaka Mukumoto, Yukinori Matsuo, and Takashi Mizowaki conceived the study, participated in its design and coordination, and helped draft the manuscript. All authors read and approved the final manuscript.

## CONFLICT OF INTEREST

We have no conflicts of interest to disclose.

## Data Availability

The data that support the findings of this study are available from the corresponding author upon reasonable request.
